# Long distance (>20 km) downstream detection of endangered stream frogs suggests an important role for eDNA in surveying for remnant amphibian populations

**DOI:** 10.7717/peerj.12013

**Published:** 2021-09-27

**Authors:** Cecilia Villacorta-Rath, Conrad J. Hoskin, Jan M. Strugnell, Damien Burrows

**Affiliations:** 1 Centre for Tropical Water and Aquatic Ecosystem Research (TropWATER), James Cook University, Townsville, QLD, Australia; 2 College of Science and Engineering, James Cook University, Townsville, QLD, Australia; 3 Centre for Sustainable Tropical Fisheries and Aquaculture, College of Science and Engineering, James Cook University, Townsville, QLD, Australia

**Keywords:** eDNA transport, Environmental DNA, Endangered species, Monitoring, Precipitation, Tropics

## Abstract

**Background:**

Globally, amphibian species have suffered drastic population declines over the past 40 years. Hundreds of species are now listed as Critically Endangered, with many of these considered “possibly extinct”. Most of these species are stream-dwelling frogs inhabiting remote, montane areas, where remnant populations are hard to find using traditional surveys. Environmental DNA (eDNA) could revolutionize surveys for ‘missing’ and endangered amphibian populations by screening water samples from downstream sections to assess presence in the upstream catchments. However, the utility of this survey technique is dependent on quantifying downstream detection probability and distances.

**Methods:**

Here we tested downstream detection distances in two endangered stream frogs (*Litoria lorica* and *L. nannotis*) that co-occur in a remote stream catchment in north-east Australia, and for which we know precise downstream distributional limits from traditional surveys. Importantly, the two last populations of *L. lorica* persist in this catchment: one small (~1,000 frogs) and one very small (~100 frogs). We conducted eDNA screening at a series of sites kilometers downstream from the populations using precipitation from two fixed water volumes (15 and 100 mL) and *via* water filtering (mean 1,480 L).

**Results:**

We detected *L. nannotis* and the small *L. lorica* population (~1,000 frogs) at most sampling sites, including 22.8 km downstream. The filtration method was highly effective for far-downstream detection, as was precipitation from 100 mL water samples, which also resulted in consistent detections at the far-downstream sites (including to 22.8 km). In contrast, we had limited downstream detection success for the very small *L. lorica* population (~100 frogs).

**Discussion:**

The ecological aspects of our study system, coupled with thorough traditional surveys, enabled us to measure downstream eDNA detection distances with accuracy. We demonstrate that eDNA from a small population of approximately 1,000 frogs can be detected as far as 22.8 km downstream from the population. Water filtration is considered best for eDNA detection of rare aquatic species—indeed it was effective in this study—but we also achieved far-downstream detections when precipitating eDNA from 100 mL water samples. Collecting small water volumes for subsequent precipitation in the lab is more practical than filtration when surveying remote areas. Our downstream detection distances (>20 km) suggest eDNA is a valuable tool for detecting rare stream amphibians. We provide recommendations on optimal survey methods.

## Introduction

Amphibians contain a greater proportion of Critically Endangered and Endangered species than any other Class of animal (IUCN, 2020). Of particular concern is that 587 amphibians are listed as Critically Endangered, which is double the number of Critically Endangered mammals, birds or reptiles (IUCN, 2020). These species are on the brink of extinction; indeed, the persistence of many is uncertain (Scheele et al., 2019; Stuart et al., 2004). Within the amphibian species listed as Critically Endangered, 143 are categorized as “possibly extinct” (CR[PE]) or “possibly extinct in the wild” (CR[PEW]) (IUCN, 2020). It is not possible to enact conservation measures for these species without knowing if, or where, they persist, and conservation actions for known Critically Endangered species are often limited by uncertainty regarding how many populations remain (Gillespie et al., 2020).

A key threat to amphibians is chytridiomycosis disease, which has particularly impacted montane, stream-associated species in the tropics (Scheele et al., 2019; Stuart et al., 2004). In the last three decades, globally, hundreds of such species have been reduced to small remnant populations or are ‘missing’ due to this disease (Scheele et al., 2019). Traditional frog surveys are typically carried out at night, when most frogs are active, and involve walking along a stream, using a head-torch to find frogs directly or *via* eye-shine (*e.g*., Puschendorf et al., 2011). Montane stream environments are a challenge for traditional surveys due to the remote, rugged terrain and seemingly countless small tributaries to search. Further, the activity of many species is dependent on weather (*e.g*., rain), impacting the probability of detection on any one survey (Scheele & Gillespie, 2018). The chances of rediscovering a small population on one section of stream (*e.g*., Puschendorf et al., 2011) are akin to ‘finding a needle in a haystack’. On the other hand, these environments offer a theoretically ideal scenario for using environmental DNA (eDNA) for threatened species monitoring. This is because the myriad tributaries flow downstream into a few major drainages, carrying eDNA from the species living in upstream habitats, including elusive and rare species (Deiner et al., 2016; Sasso et al., 2017). However, the use of eDNA screening to survey entire catchments, or parts of a catchment, relies on knowledge of maximum downstream detection distances of a target species.

Downstream eDNA detection distance depends on multiple factors, such as eDNA shedding rate, decay, eDNA displacement, retention and resuspension (Barnes, Turner & Turner, 2016), as well as population abundance (Yates, Fraser & Derry, 2019). Cage experiments are particularly pertinent to understanding the downstream detection of small population sizes (*i.e*., equivalent of remnant frog populations) because small numbers of individuals are used and downstream limits are known. Cage experiments using up to 50 individuals of the target species show limited detection distances, typically in the order of hundreds of meters (Table S1). A limitation of cage studies is that they typically use very small numbers of individuals (Schumer et al., 2019; Table S1). The exception is Laporte et al. (2020), who tested downstream detection of a high biomass of fish (49 individuals, 28 kg total biomass) and found one positive detection five kilometers downstream from the cage. Another limitation of cage studies is that the target organism is typically in the environment for only a short period prior to sample collection (*i.e*., no accumulation of eDNA in the environment) (Schumer et al., 2019).

Similarly, most studies of downstream eDNA detection of wild populations have shown short detection distances, ranging from hundreds of meters to less than five kilometers (Civade et al., 2016; Jane et al., 2015; Wilcox et al., 2016). Only a few studies have shown downstream detection distances greater than ten kilometers, and these ‘far-downstream’ detection distances have been for species that were either abundant at the source (Deiner & Altermatt, 2014; Itakura et al., 2019; Pont et al., 2018) or species for which the downstream limit was not accurately known (Deiner & Altermatt, 2014; Pont et al., 2018), and hence downstream detection distances may have been over-estimated. Few studies have assessed downstream detection distance of small wild populations. Environmental DNA detection of a very small freshwater pearl mussel aggregation (100 individuals) was limited to immediately downstream from the population, compared with up to 1.7 km downstream from a larger aggregation of more than 10,000 individuals (Wacker et al., 2019). Another study on freshwater pearl mussels did not detect eDNA more than 25 m downstream from a population of up to 20,000 individuals (Stoeckle, Kuehn & Geist, 2016).

Many studies have used eDNA to detect upstream amphibian populations (Bedwell & Goldberg, 2020; Lopes et al., 2020; Pilliod et al., 2014; Santas et al., 2013; Sasso et al., 2017; Spear et al., 2015). For example, Santas et al. (2013) and Spear et al. (2015) used targeted eDNA surveys to detect Hellbender salamanders (*Cryptobranchus alleganiensis*) at known, and previously unknown, sites. Sasso et al. (2017) used a metabarcoding approach to detect amphibian communities across four streams from separate drainages in the Brazilian Atlantic rainforest. They showed that a 4-day eDNA sampling event captured all stream species found during a 5-year long survey using traditional techniques. These and other studies to date have shown that eDNA surveys are efficient for amphibian species detection, but did not determine downstream detection distances, primarily because the lowest distributional limits of species were not known.

Environmental DNA could revolutionize the way we survey for ‘missing’ and critically endangered amphibians (and, potentially, other stream taxa) (Ficetola, Manenti & Taberlet, 2019). Most of these amphibian species are in upland areas, using small headwater streams, but eDNA could be transported from these into a small number of large streams that flow off the mountains. Access to the myriad upland streams is typically limited, but the large streams at the base of mountains are usually crossed by roads, allowing easy sampling points for collecting water. Detections in large streams would then focus further eDNA sampling or traditional field survey efforts in upstream areas of likely habitat to more efficiently locate populations. However, the utility of eDNA is dependent on demonstrating significant downstream eDNA detection distances of amphibians, particularly endangered species with small and localised populations.

Rainforest stream frogs inhabiting the mountains of eastern Australia have been heavily impacted by chytridiomycosis disease (Scheele et al., 2017). Declines started in the late 1970s near Brisbane (south-east Queensland) and progressed north to impact frogs of the Wet Tropics World Heritage Area, in north-east Queensland, in the late 1980s and early 1990s (Laurance, McDonald & Speare, 1996; Scheele et al., 2017). A total of six species are believed to have gone extinct in this period, while other species declined substantially and now persist in a fraction of their former range (Scheele et al., 2017). The persistence of several species remains uncertain because they have not been seen for two or three decades and surveys have not been conducted in remote, rugged parts of their former ranges (Hoskin & Puschendorf, 2014; Gillespie et al., 2020; Meyer et al., 2020). For the same reason, the number of populations of several Critically Endangered species is not known, and locating these populations has been identified as a priority research action (Gillespie et al., 2020).

### Study system and aims

The Armoured Mistfrog (*Litoria lorica*) is a Wet Tropics species that was considered to be extinct after severe chytridiomycosis-related declines in the early 1990s (Cunningham, 2002; Puschendorf et al., 2011). However, it was rediscovered as a single, small population in 2008 during research on populations of a co-occurring Endangered species, the Waterfall Frog (*Litoria nannotis*) (Puschendorf et al., 2011). These two frogs differ in size (*L. lorica* approximately 37 mm; *L. nannotis* approximately 55 mm long) but have near-identical ecologies, foraging side-by-side at night in the splash zone of waterfalls and cascades, and hiding by day in rock cracks in the flowing water (Puschendorf et al., 2011). The eggs and tadpoles of both species are also restricted to the same fast flowing sections, with the tadpoles of both species having suctorial mouth discs (Anstis, 2013; Puschendorf et al., 2011; C. Hoskin, 2021, unpublished data). Therefore, both species are semi-aquatic inhabitants of a very specific habitat—waterfalls and cascades.

**Figure 1:**
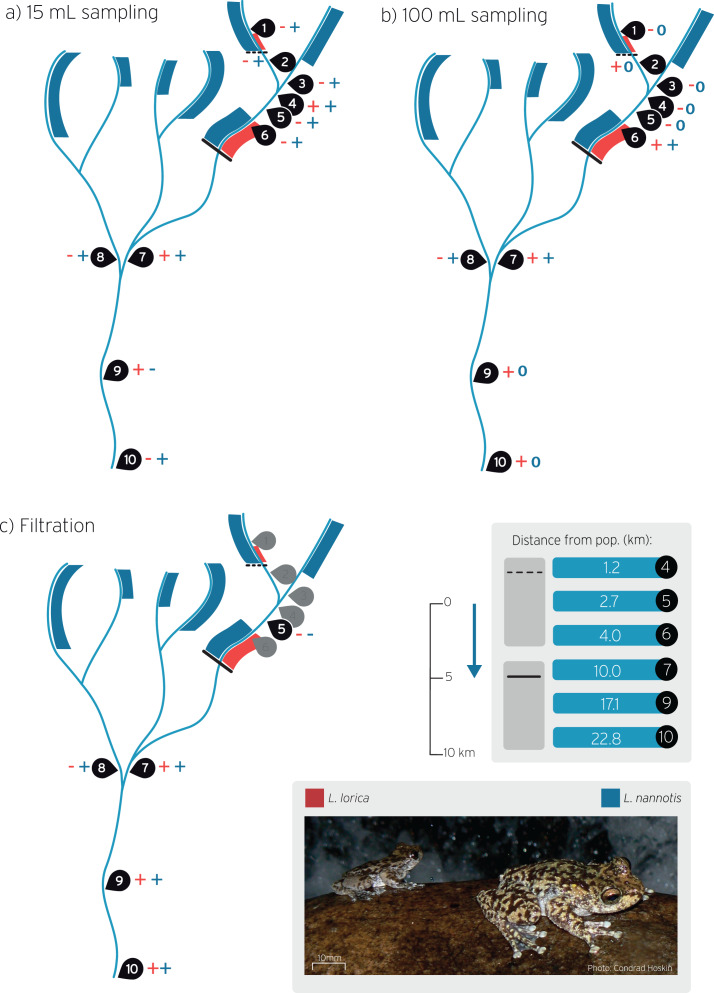
Stream sites sampled for *L. lorica* and *L. nannotis* eDNA detection during wet season sampling. Water sampling was carried out using the: (A) The 15 mL sampling method, (B) The 100 mL sampling method, and (C) on-site filtration method. Sampling sites are numbered 1–10. Red and blue bands on streams show the distribution of *L. lorica* and *L. nannotis*, respectively, determined using traditional surveys. Size of bands denote approximate population sizes estimated from traditional surveys. The dashed line and the solid black line denote the downstream limit of the reintroduced and main population of *L. lorica*, respectively. The table shows the distance from the dashed and solid lines to downstream sampling sites. Plus symbols (+) show sites of positive detection for *L. lorica* (red) and *L. nannotis* (blue). Minus symbols (−) show no detections for that species (see Table 2 for details). The blue 0 symbols on panel b show the sites where no data was obtained for *L. nannotis* due to qPCR machine failure. The blue arrow shows stream flow direction.

The rediscovery of *L. lorica* triggered on-ground surveys of most potential habitat in the region, including surveying streams throughout the large catchment where *L. lorica* was rediscovered (Hoskin & Puschendorf, 2014; Hoskin & Puschndorf, 2021, in preparation) (Fig. 1). These surveys found populations of *L. nannotis* in all sections of suitable habitat but did not find any additional populations of *L. lorica*. Permission was obtained for a trial reintroduction of *L. lorica*, which involved translocating adults during three consecutive years (2013–2015) to establish a population in a discrete area of suitable habitat approximately 4 km upstream of the rediscovered population (Hoskin & Puschendorf, 2014; Hoskin & Puschendorf, 2021, in preparation) (Fig. 1). Based on regular monitoring of these two populations over the last five years, the rediscovered population is estimated to consist of approximately 1,000 frogs (plus an unknown number of aquatic tadpoles at any one time) along a 4 km stretch of stream, and the reintroduced population is estimated at about 100 frogs (and a small number of tadpoles at any one time) along an approximately 1 km section of stream (Hoskin & Puschendorf, 2014; Hoskin & Puschendorf, 2021, in preparation) (Fig. 1).

Here we aimed to test far-downstream distance eDNA detection of *L. lorica* and *L. nannotis* in order to determine the utility of eDNA as a method for surveying endangered stream frogs. We used three eDNA sampling methods (15 and 100 mL water volumes for precipitation, and large volume filtering of >1,000 L) to determine downstream detection at a series of sites kilometers downstream of the resolved lower limits of *L. lorica* and *L. nannotis*. This system is ideal for testing downstream detection distances for the following reasons. First, adults of the two species have daily contact with water, so eDNA can enter the stream at all times regardless of the presence of tadpoles. Second, the catchment containing *L. lorica* has been thoroughly surveyed (*i.e*., on foot, using head torches at night), including all areas of potentially suitable habitat for these two species (Hoskin & Puschendorf, 2014; Hoskin & Puschendorf, 2021, in preparation). These surveys have resolved downstream limits for the two species on all sections of streams (Fig. 1; details below). Third, *L. nannotis* is present and common in discrete upstream sections of all streams, whereas *L. lorica* is restricted to two sections—the small main population (approximately 1,000 frogs) and the very small reintroduced population (approximately 100 adults). We were therefore able to test eDNA detection at accurately calculated downstream distances from both species, and from both populations of *L. lorica*. We use these results to outline the feasibility of eDNA for detecting small upstream populations of stream frogs.

## Materials and Methods

### Summary of traditional field surveys

The downstream limits for *L. lorica* and *L. nannotis* have been determined in detail in this catchment. Field surveys have been conducted in all areas of suitable habitat, specifically targeting *L. lorica*, *L. nannotis* and other ‘missing’ and endangered stream frogs (Hoskin & Puschendorf, 2014; Puschendorf et al., 2011; C. Hoskin, 2021, unpublished data). In particular, the objective of the surveys was to locate populations of stream frogs in the lower sections of each stream, based on the hypothesis that these areas are refuges from chytrid disease impacts due to warmer conditions associated with lower elevation and open canopy woodland (Hoskin & Puschendorf, 2014; Puschendorf et al., 2011). Some of these areas have been surveyed repeatedly over the last decade to confirm presence/absence at lower and upper range limits, and monitor numbers along key stream sections (Hoskin & Puschendorf, 2021, in preparation). *Litoria lorica* and *L. nannotis* require steep, rocky, permanently flowing streams. Adults of both species are readily located in surveys due to their restriction to waterfalls and cascades, and due to their reliable detection when present at a site (Puschendorf et al., 2011). All surveys were done by two highly experienced frog biologists (one of which was always C. Hoskin).

The basic procedure for field surveys was to locate the downstream limits for *L. lorica* and *L. nannotis* and then survey upstream to the rainforest. Suitable stream sections were identified from Google Earth imagery, accessed by helicopter or car, and then surveyed on foot. The streams in this region have a predictable morphology. Each is sourced above 1,100 m in the rainforest uplands. The stream aspect is initially low in the uplands, and then grade increases substantially as the stream flows out of the rainforest around 900 m elevation and steeply descends through fairly continuous (and often substantial) waterfalls and cascades on exposed granite bedrock through open canopy forest, down to about 500 m elevation, where each stream then abruptly flattens out. This abrupt transition from steep to relatively flat marks the end of suitable habitat for these species. Stream gradient is then consistently very low, and the stream consists of long pools separated by short, sandy riffle areas. Surveys extended to the lowest area of suitable habitat on each stream (*i.e*., the lowest waterfall/cascade), which is between 450 and 500 m elevation, and beyond for some distance along the flat stream. *Litoria lorica* and *L. nannotis* were never found beyond the lowest waterfall or cascade (Hoskin & Puschendorf, 2014; Hoskin & Puschendorf, 2021, in preparation). Some stream sections kilometers downstream from the downstream limit of suitable habitat for *L. lorica* and *L. nannotis* were also surveyed to confirm absence of these species. The results of surveys in this catchment are summarized in Fig. 1, showing the distribution of *L. lorica* (red) and *L. nannotis* (blue) on each stream section.

### Stream water sampling

The present study was conducted in a permanently flowing stream catchment in the Wet Tropics of Queensland, north-east Australia. Tropical Australia exhibits high rainfall seasonality (Feng, Porporato & Rodriguez-Iturbe, 2013; Lough, 1993), with a wet season (generally December–April) and a dry season (generally May–November). This seasonality is evident in the monthly rainfall for the study region in 2018–2019 (Fig. S1). This study included two eDNA sampling trips. The most comprehensive was conducted during the wet season (8–10 April 2019) and included all sites shown in Fig. 1. These are: a site immediately downstream from the reintroduced *L. lorica* population (site 2) and then two sites further downstream from this population (sites 4, 5); a site about 10% into the main *L. lorica* population (site 6); three sites (sites 7, 9, 10) far-downstream from the main *L. lorica* population (10.0, 17.1 and 22.8 km, respectively); and three sites in tributaries where only *L. nannotis* has been found upstream in field surveys (sites 1, 3, 8) (Fig. 1). Site 2 (immediately downstream from the reintroduced population) and site 6 (at the upper end of the main *L. lorica* population) are comparable because there is an estimated 100 adult *L. lorica* immediately upstream from both sites (C. Hoskin, 2019, unpublished data). All sites and all three eDNA sampling methods (outlined below) were done on this trip.

A dry season sampling trip was also conducted to assess whether far-downstream detection was still possible during reduced stream flow. Sampling during the dry season was conducted on 24–25 October 2019, and only included a subset of the sites (sites 6–10) deemed most informative for comparing long distance eDNA transport. These sites were the four far-downstream sites (7–10), and site 6 as a ‘positive control’ (*i.e*., sampling water where both species occur). Environmental DNA sampling during this trip was only conducted using the 100 mL sampling method (see below), with five replicates taken at each site (Table S2). Water volume in the stream during this dry season sampling trip was observed to be markedly less than during the wet season trip, as seen in comparative photos at site 7 (Fig. S1).

### Environmental DNA field sampling methods

We used three eDNA sampling methods during the wet season sampling event: (1) direct water collection and preservation of 15 mL samples; (2) direct water collection and preservation of 375 mL samples (from which 100 mL was subsequently sub-sampled); and (3) on-site filtration of large volumes of water. The different methods were used to ultimately assess far-downstream detection success against feasibility of sampling remote populations (*i.e*., amount and weight of gear/samples carried on foot).

**Figure 2:**
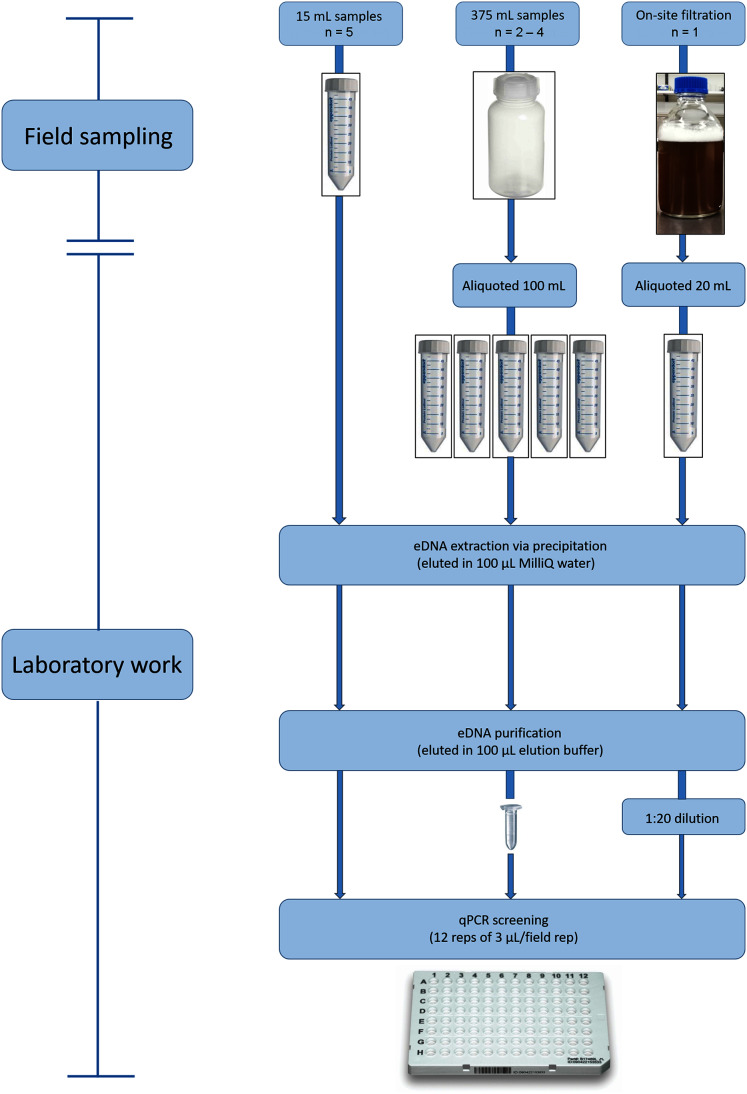
Environmental DNA workflow for the wet season sampling from eDNA collection through to qPCR screening for *L. lorica* and *L. nannotis*. We used three water screening volumes/methods: ‘15 mL’ (left), ‘100 mL’ (middle), and large volume ‘filtration’ (right). During dry season sampling, we only used the 100 mL method and followed the laboratory workflow shown above for that method. The number of field replicates collected for each sampling method is shown as ‘*n* = ’.

For the 15 mL water sampling method, a new 50 mL Falcon tube was used to decant a 15 mL sample of stream water into another 50 mL Falcon tube containing ten mL of Longmire’s preservative solution (Longmire, Maltbie & Baker, 1997) at the Longmire’s: water ratio used by Edmunds & Burrows (2020) (Fig. 2). A total of five replicates of the 15 mL samples were taken in close proximity at each site (Table 1). Additionally, a field blank (control) was taken at each site, by conducting the same procedure but decanting 15 mL of MilliQ water rather than stream water into the tube containing the Longmire’s solution.

**Table 1:** Water sampling details.

Site	15 mL samples			100 mL samples			Filtration		
	# field reps	# technical reps	Volume (mL)	# field reps	# technical reps	Volume (mL)	# field reps	# technical reps	Volume (mL)
1	5	60	75	2	24	150	–	–	–
2 (translocated)	5	60	75	2	24	150	–	–	–
3	5	60	75	2	24	150	–	–	–
4	5	60	75	2	24	150	–	–	–
5	5	60	75	4	48	300	1	12	1,614,000 × 10^3^
6 (main pop)	5	60	75	3	36	225	–	–	–
7	5	60	75	4	48	300	1	12	1,458,000 × 10^3^
8	5	60	75	4	48	300	1	12	1,805,000 × 10^3^
9	5	60	75	4	48	300	1	12	1,377,000 × 10^3^
10	5	60	75	4	48	300	1	12	1,145,000 × 10^3^

**Note:**

Number of field replicates, technical qPCR replicates, and volume of water processed (mL) per eDNA capture method at each sampling site during the wet season sampling. The filtration method lacks data for some cells because it was only conducted at five key sites.

The 100 mL sampling method was performed by using a new bottle of 500 mL capacity to take a 375 mL sample from the stream and decant it into a 500 mL bottle containing 125 mL of Longmire’s preservative solution (Fig. 2). A field blank was included for each site, by decanting 375 mL of MilliQ water into a 500 mL bottle containing 125 mL of Longmire’s solution. Between two and four replicate 375 mL stream water samples were collected at each site, depending on how far the walk was to sites (*i.e*., the limitation being carrying water sample weight over long distances) (Table 1). A total of two replicate 375 mL samples were collected at sites 1–4, three replicates at site 6, and four replicates at site 5 and sites 7–10 (Table S2; Table 1). In the laboratory, each 375 mL sample was decanted into five aliquots of 20 mL each for eDNA extraction (Fig. 2). Therefore, a total of 100 mL of each 375 mL replicate was screened for eDNA and hence this method is hereafter termed the ‘100 mL sampling method’. Field work was conducted under Queensland Government permit WITK18662017 (to C.J.H).

For the on-site filtration method, water was filtered through a deep filter layer of 1 µm nominal pore size, using a sampler system described by Stevens (2020) and used by Lewis et al. (2018). A flow meter was attached to record the volume of water processed. Filtering was conducted at five sites with relatively close road access because this filtering device was large and heavy (approximately 5 kg). A single replicate was collected at each site, consisting of water filtration for a period of 1 h. The mean total volume of filtered water across the five sites was 1,480 L (± 111.11 SE) (see Table 1 and Table S2 for filtered volumes at each site). After filtration was completed, the filter was preserved in 700 mL of Longmire’s solution diluted to 25% in MilliQ water. An equipment blank was included for each site, which involved preserving a new, clean filter in 700 ml of Longmire’s buffer diluted with water to 25%, with the procedure conducted on-site. In order to allow the eDNA in the filters to resuspend, filters in Longmire’s solution were stored indoors at room temperature for 1 week after filtration. After this period, each water sample was mixed by inversion ten times in order to ensure that all eDNA was resuspended before taking an aliquot for extraction. A 20 mL aliquot from the diluted Longmire’s solution in which the filter had been preserved was then taken and eDNA was extracted (Fig. 2).

### Environmental DNA extractions

Upon arrival to the laboratory, eDNA was extracted *via* a glycogen-aided isopropanol based precipitation protocol (Villacorta-Rath et al., 2020) in a dedicated eDNA laboratory at James Cook University (JCU), Australia. For all extractions, 20 mL sample aliquots were mixed with 5 µL glycogen (200 mg/mL), 20 mL isopropanol and 5 mL NaCL (5M). Samples were then incubated overnight at 4 °C and subsequently centrifuged at 6,750 g for 10 min to form a pellet. The supernatant was then discarded and pellets were dissolved in 600 µL of lysis buffer (guanidinium hydrochloride and TritonX; pH 10), transferred into a 2 mL DNA LoBind^®^ Tube (Eppendorf), and frozen for up to four months at −20 °C to enable processing all samples as soon as possible. Environmental DNA present in the samples was lysed at 50 °C for 5 h and a subsequent precipitation step was carried out by adding 1 µL glycogen and 1,800 µL polyethylene glycol (PEG) buffer to the samples. Samples were centrifuged at 20,000 g for 30 min to form a pellet that was then washed twice using 70% ethanol. After the ethanol washes, the pellet was dried and eDNA was resuspended in 100 µL MilliQ water. Finally, eDNA was purified using the Qiagen DNeasy^®^ PowerClean^®^ Pro Cleanup kit and eluted in 100 µL elution buffer. Given that we sampled very large volumes of water with our filtration system, samples were highly pigmented (Fig. S2). After eDNA extraction and purification, samples still contained a high level of coloration and therefore we applied a 1:20 dilution to result in a sample that did not exhibit any coloration. We applied inhibition tests (Jane et al., 2015) on the final 1:20 dilution.

Screening of the 100 mL samples from the wet season sampling was complicated by a problem in the laboratory. A mechanical failure of the real-time PCR machine during screening of *L. nannotis* from sites 1–5 and 9–10 resulted in complete failure of those samples. Re-extraction from those water samples was conducted 5 months later but this resulted in minimal detections. Given the high detection frequencies of sites 6, 7 and 8 (27–46%; Table 2), which were successfully screened during the first round of qPCR, it was concluded that the eDNA had degraded in the intervening months. This was despite the fact that the samples were mixed with Longmire’s solution and stored at room temperature in the dark, which had been demonstrated to protect eDNA from degradation for at least 8 weeks (Edmunds & Burrows, 2020).

**Table 2:** Percentage of *Litoria* species positive detections per eDNA capture method during the wet season sampling.

Site	15 mL samples		100 mL samples		Filtration	
	*L. lorica*	*L. nannotis*	*L. lorica*	*L. nannotis*	*L. lorica*	*L. nannotis*
1	0	8.3	0	–	–	–
2 (translocated)	0	43.3	12.5	–	–	–
3	0	1.6	0	–	–	–
4	1.7	28.3	0	–	–	–
5	0	3.3	0	–	0	0
6 (main pop)	0	25.0	13.9	44.4	–	–
7	3.3	3.3	37.5	45.8	100	66.7
8	0	3.3	0	27.1	0	91.6
9	3.3	0	16.7	–	50.0	83.3
10	0	5.0	12.5	–	17.0	25.0

**Note:**

The number in each data cell represents the percentage of technical replicates that were positive. There was no data for *L. nannotis* for the 100 mL sampling method from sites 1–5 and 9–10 due to a mechanical failure of the qPCR machine. The filtration method lacks data for sites 1–4 and 6 because it was only conducted at five key sites.

### Real-time PCR (qPCR)

We used two different species-specific primer pairs targeting the cytochrome *c* oxidase subunit I (COI) mitochondrial gene of *L. lorica* and *L. nannotis* (Table S3; Edmunds et al., 2019). The limit of detection (LOD) was estimated using a ten-fold serial dilution of double-stranded synthetic DNA fragments (qBlocks^TM^ Integrated DNA Technologies Pty Ltd., New South Wales, Australia) synthesized to match the target fragments of each of the study species, ranging from 2.82 × 10^7^ to 0.70 copies/µL for *L. lorica* and 2.17 × 10^7^ to 0.55 copies/µL for *L. nannotis* (Edmunds et al., 2019). Additionally, the LOD was determined using a seven-fold serial dilution of genomic DNA (gDNA) derived from toe pad tissue from each of the target species. For *L. lorica*, gDNA dilutions ranged between 1.24 and 3.1 × 10^−5^ ng/μL, and between 6.45 × 10^−4^ and 1.6125 × 10^−5^ ng/μL for *L. nannotis* (Edmunds et al., 2019). Between four and six technical replicates per dilution were used, and the LOD was set at the lowest standard with 95% or greater detection (Klymus et al., 2019). Based on the serial dilutions, the LOD was determined to be 4.9 × 10^−5^ ng/μL or 2 copies/reaction for *L. lorica*, and 4.8 × 10^-5^ ng/μL or 2 copies/reaction for *L. nannotis*.

qPCR assays were performed on a QuantStudio™ 3 or QuantStudio™ 5 Real-Time PCR System (Thermo Fisher Scientific, Scoresby, VIC, Australia Pty Ltd) in white 96 or 384-well plates, respectively, and sealed with optical films (Thermo Fisher Scientific, Scoresby, VIC, Australia Pty Ltd). Presence of *L. lorica* and *L. nannotis* was screened through twelve technical qPCR replicates of each field replicate, representing 36% of the total available DNA elution volume for each species. This is a much higher number of technical replicates than most eDNA studies use (reviewed by Rees et al., 2014). High field replication is needed in order to avoid false negative detections (Furlan et al., 2016), so by thoroughly screening each field replicate we maximised the chances of detecting the target species eDNA in the available replicate samples. The downside of increasing the number of technical replicates is that the false positive rate also increases; however, screening field and extraction controls, and qPCR controls, can account for this problem (Bustin et al., 2009). Additionally, a triplicate positive control consisting of gDNA of the target species and three no-template controls (NTC) was used (Bustin et al., 2009). The NTC samples did not contain the target species DNA and their lack of amplification indicated that no contamination was introduced during plate handling. Each qPCR assay consisted of 3 µL of template DNA and 7 µL of master mix (5 µL PowerUp SYBR Green Master Mix; 0.5 µL forward primer at 10 µM; 0.5 µL reverse primer at 5 µM; 1 µL MilliQ^®^ water). Thermal cycling conditions were as follows: initial denaturation and activation at 95 °C for 2 min, then 55 cycles of 95 °C for 15 s, and 60 °C for 1 min. A subsequent melt curve analysis was performed to generate dissociation curves by transitioning from 60 to 95 °C, at 0.15 °C s^−1^.

All plates were analysed with a common fluorescence threshold of 0.2 using QuantStudio™ Design and Analysis Software (version 1.4.2; Thermo Fisher Scientific, Scoresby, VIC, Australia Pty Ltd.) before export and subsequent analyses in Microsoft Excel. Samples were considered putative positive detections if: (1) the amplification curve crossed the common fluorescence threshold within 50 cycles; (2) the amount of eDNA was above the LOD; and (3) the melt curve analysis showed a dissociation temperature peak at 78.52 °C (±0.62–99% confidence interval) for *L. lorica* and 79.66 °C (±0.75–99% confidence interval) for *L. nannotis*. Amplicons from putative positive detections were sequenced *via* dual direction Sanger sequencing at the Australian Genome Research Facility (AGRF) to confirm that they were true detections. Amplicon sequences from the samples considered putative positive detections were considered as true detections if there was ≥99% pairwise identity to in-house *L. lorica* sequences (no sequences on GenBank), with the targeted *COI* section being: CGACACTTATTATGTTGTAGCCCATTTCCATTATGTATTGTCTATAGGAGCTGTATTCGCCATTATAGC (Edmunds et al., 2019). This section of *L. lorica* sequence is invariant among *L. lorica* sequences and has at least three mismatches to other frog species. For *L. nannotis*, positive detections were considered true detections if they exhibited ≥97% pairwise identity with the *COI* gene of the species (GenBank accession number JN130908 matching between positions 219–286 bp; accession number JN30913 matching between positions 167–286 bp; in-house sequences).

### Inhibition test

We tested inhibition in water samples by spiking 80 copies of artificial DNA into triplicated samples from the sites without presence of *L. lorica* (sites 1, 3 and 8) and 8 copies of artificial DNA into all field controls. Additionally, we spiked the same number of DNA copies into three technical replicates containing only MilliQ water. A sample was considered inhibited if it exhibited a shift in Ct values of three or more cycles when compared to the spiked MilliQ water (Cao et al., 2012).

### Calculating detection frequencies and downstream detection distances

Detection frequency for each species was calculated as the proportion of qPCR replicates of each sampling method at each site that yielded a positive detection, in relation to the total number of qPCR replicates at that site. Detection frequencies for each eDNA capture method were calculated separately for the two sampling trips. Standard errors of these proportions were calculated by dividing the standard deviation of the number of positive qPCR detections by the square root of the total number of qPCR replicates (Calter & Calter, 2011). Downstream detection distances were calculated by using the path distance function in Google Earth to measure the distance from the downstream limit of the species on each stream (from field surveys) to the eDNA sampling site. Of particular relevance were the downstream distances from the two populations of *L. lorica* (Fig. 1).

The primary aim of this study was to determine downstream detection distances for each species, with a particular focus on far-downstream distances (*i.e*., >10 km, >20 km). Beyond this overall aim, we could also assess determinants of detection success where sufficient data was obtained. The three determinants assessed were: (1) whether precipitation from larger water volumes (*i.e*., 100 mL *vs*. 15 mL) yielded higher detection frequencies in *L. lorica* wet season samples; (2) whether detection frequencies were higher for the considerably more abundant species, *L. nannotis*; (3) whether there was higher probability of detecting *L. lorica* during the wet season than during the dry season. The methods for assessing these are described in the sections below. Other determinants of detection success could not be assessed due to insufficient numbers of comparisons. This was primarily due to the mechanical failure of the real-time PCR machine, which impacted *L. nannotis* wet season screening for the 100 mL sampling method. This precluded testing for higher detection frequencies for the 100 mL *vs*. 15 mL method for *L. nannotis*, or testing for higher detection frequencies for *L. nannotis vs. L. lorica* using the 100 mL sampling method. The water filtering technique was used at too few sites to test for differences in detection success compared to the 100 mL and 15 mL precipitation methods.

#### Did precipitation from larger water volumes yield higher detection frequencies?

There was sufficient data from the *L. lorica* wet season sampling to test the prediction that the 100 mL sampling method produces more detections than the 15 mL method. This prediction is based on the fact that about five times as much water was screened in the 100 mL sampling method *vs*. the 15 mL method at each site. This was tested using a Wilcoxon signed-rank test, using the paired detection frequencies for the two water sampling methods at the seven sites where *L. lorica* could have been detected (*i.e*., sites 2, 4–7, 9, 10) (Fig. 1).

Additionally, occupancy models were fitted to the eDNA detection data (Supplemental Informations 2 and 3) using the ‘eDNAoccupancy’ R package (Dorazio & Erickson, 2018). This package fits Bayesian occupancy modelling to nested data commonly used in eDNA surveys, taking into account the sampling sites (primary sample units), the samples collected within each site (secondary sample units) and the qPCR technical replicates of each sample at each site (replicate observations) (Dorazio & Erickson, 2018). To model the probability of detection in the 100 and 15 mL samples, we considered that eDNA detection depends on distance from the population, estimated upstream population abundance, and volume of water processed (Supplemental Informations 2 and 3). The model was fitted using the *occModel* function, with MCMC chains run for 11,000 iterations, with 10,000 retained for parameter and confidence interval estimation.

### Was the more abundant species more readily detected?

We had sufficient data from the 15 mL wet season sampling to test the prediction that *L. nannotis* would be more readily detected than *L. lorica*. This prediction was based on the fact that there is estimated to be at least an order of magnitude more *L. nannotis* upstream of all these sites (Table S5). The difference in detection frequencies for the two species was tested using a Wilcoxon signed-rank test, comparing the detection frequencies for each species at sites where both species could both have been detected (*i.e*., sites 2, 4–7, 9, 10; Fig. 1). Occupancy models were also run to estimate the probability of detection of each species during the 15 mL wet season sampling. The modelling was conducted as described above, but with ‘species’ added as a covariate (Supplemental Informations 2 and 3).

### Was detection probability higher in the wet season than the dry season?

The wet and dry season sampling was not designed to test for significant differences in detection frequencies with stream flow (cf. Jane et al., 2015; Shogren et al., 2018) but rather the two sampling trips were conducted to determine if far-downstream detection was still possible under low stream flow conditions of the dry season. Lower detection chances were assumed based on longer eDNA transport times to downstream sites under dry season (low flow) conditions. There were not enough comparisons to statistically test for a difference in detections between the wet and dry season sampling. However, occupancy models were run to estimate the probability of detecting *L. lorica* in the 100 mL sampling during the wet and dry seasons. The modelling was conducted as described above, but with ‘season’ added as a covariate (Supplemental Informations 2 and 3).

## Results

### Inhibition tests

Mean Ct values for all spiked field samples and field controls did not show a shift in Ct values of more than one cycle (Tables S6 and S7), indicating that no inhibition was present in the qPCR assays.

### Detection frequencies and downstream detection distances for the three methods

All positive detections satisfied the following conditions: (1) amplification occurred within 50 cycles; (2) amount of eDNA was above the LOD; (3) the melt curve peak corresponded to that of each species; and (4) amplicon sequences from positive detections had >97% and >99% match with targeted COI regions for *L. lorica* and *L. nannotis*, respectively. The qPCR of all field control samples and extraction control samples were devoid of *L. lorica* and *L. nannotis* DNA (Table S5).

**Figure 3:**
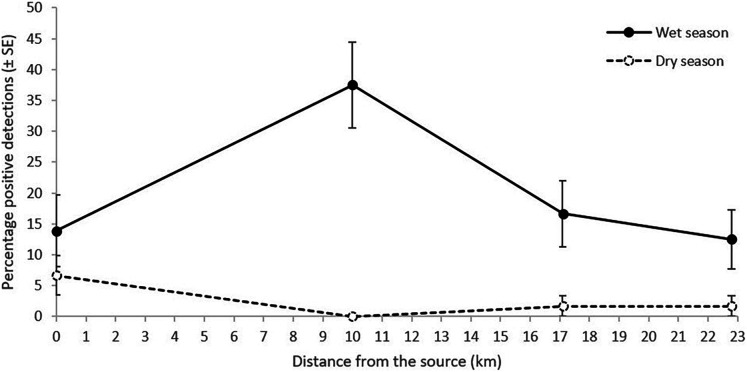
Percentage of *L. lorica* positive detections (±SE) from the technical replicates at each site from the 100 mL sampling method during the wet season (solid line) and the dry season (dashed line). The 0 km site is site 6, sampled in the main *L. lorica* population, and the other three sites are plotted by distance from the lower limit of the main *L. lorica* population (*i.e*., sites 7, 9 and 10; Fig. 1; Table S4).

Precipitation of the 15 and 100 mL samples, and water filtration gave positive detections of *L. lorica* and *L. nannotis* at most of the sampling sites where each of the species is known to occur at the site or upstream (Table 2; Fig. 1; Table S4). Of particular note are the far-downstream detections (10, 17.1 and 22.8 km) for both species using all three methods during the wet season sampling (Table 2; Figs. 1 and 3), and for the 100 mL sampling method during the dry season sampling (Fig. 3; Table S4). Importantly, we did not detect *L. lorica* eDNA at the three sites where this species is not known to occur upstream (*i.e*., sites 1, 3, 8; Table 2; Fig. 1; Table S4).

### Comparing detection success from the three eDNA sampling methods during wet season sampling

#### Filtration

Filtration was only performed during the wet season sampling trip, at the five sites with reasonably close road access. Surprisingly, filtration gave no positive detections of either species at site 5 (2.7 km downstream; Table 2). In contrast, this method yielded high detection frequencies for both species at the four far-downstream sites (*i.e*., > 10 km) (Table 2). Positive detections of *L. nannotis* eDNA ranged from 91.6% at site 8, to 25% at the furthest downstream site (10). For *L. lorica*, 100% of qPCR technical replicates showed positive detections at site 7, 10 km downstream from the main population. The positive detections then decreased with distance to 50% at site 9 (17.1 km from main population) and 17% at site 10 (22.8 km from main population). Although there was a general trend of decreasing detection frequencies with distance (Table 2), with only four sites it was not possible to analyse this statistically. The percentage of positive detections using water filtration was higher for the far-downstream sites compared to the two precipitation methods (Table 2), but the small number of filtration sites means it was not possible to test this statistically.

#### Did precipitation from larger water volumes yield higher detection frequencies?

**Figure 4:**
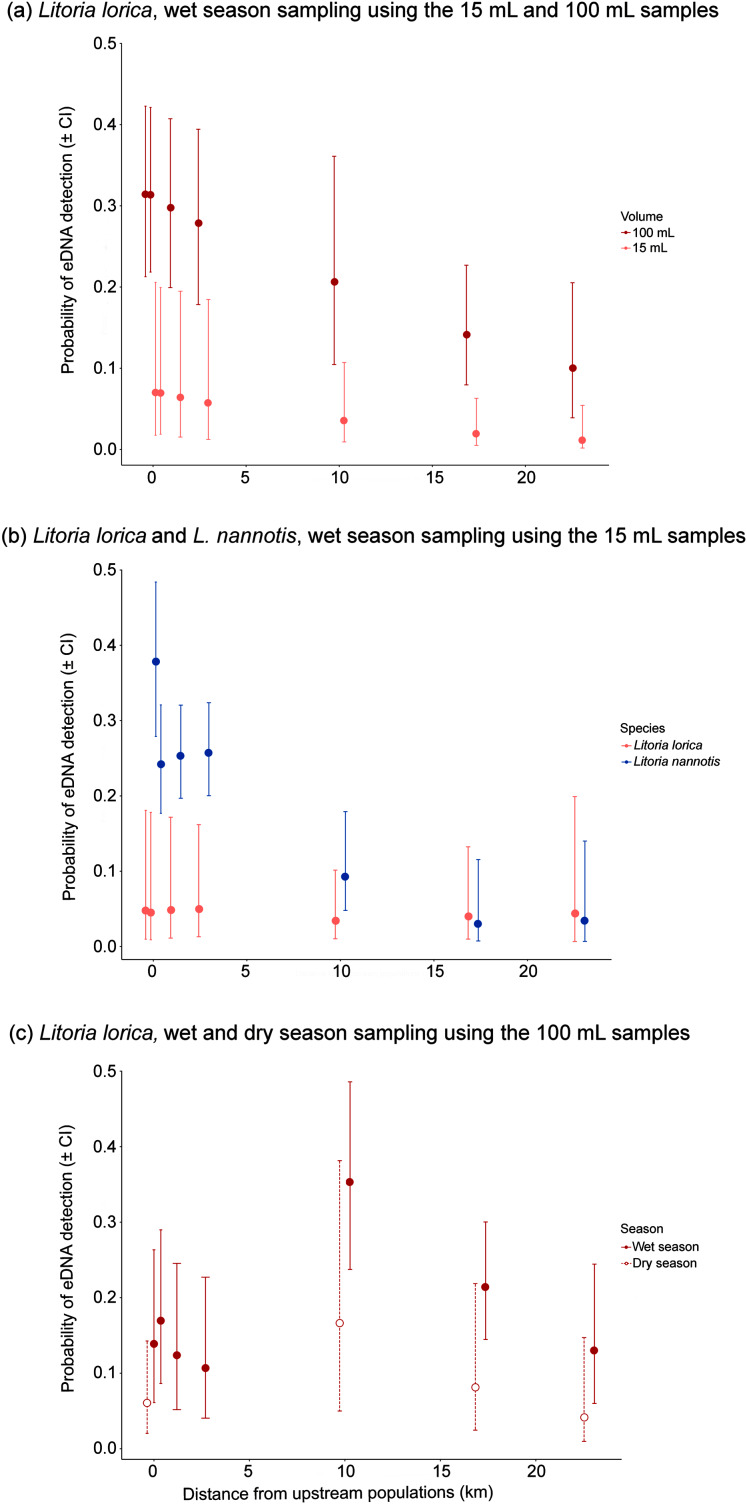
Probability of eDNA detection with downstream distance resulting from site occupancy models. Data is based on: (A) *L. lorica* wet season sampling using the 15 and 100 mL sampling methods; (B) *L. lorica* and *L. nannotis* during wet season sampling using the 15 mL sampling method; and (C) *L. lorica* wet and dry ­­season sampling using the 100 mL sampling method. Dot points represent median values and error bars are 95% confidence intervals. Sampling sites located <10 km from the upstream population had approximately 100 adult *L. lorica* upstream upstream; whereas sampling sites located 10 km or more from the upstream population had approximately 1,100 adult *L. lorica* upstream upstream (see Fig. 1; Supplemental Informations 2 and 3). All sampling sites had large populations of *L. nannotis* upstream (see Fig. 1; Supplemental Informations 2 and 3).

For the precipitation method, *L. lorica* detection frequencies were usually higher using 100 mL samples than 15 mL samples, and generally by an order of magnitude (Table 2; Fig. 4A). For example, at the furthers three sites that *L. lorica* could have been detected (sites 7, 9 and 10), percentage of positive detections using the 100 mL samples were 37.5%, 16.7% and 12.5%, respectively, *vs*. 3.3%, 3.3% and 0% for the 15 mL samples. This difference was expected based on the fact that the 100 mL sampling method screened about five times as much water at each site. A Wilcoxon signed-rank test, using the paired average probabilities for each sampling method at the seven sites where *L. lorica* could have been detected (*i.e*., sites 2, 4–7, 9, 10) (Table 2; Fig. 1), revealed the 100 mL sampling method to be significantly better for detecting *L. lorica* than the 15 mL method (mean difference 1.38, z = −1.99, W = 1, *P* < 0.05). The site occupancy model revealed higher probability of *L. lorica* detection at all sampling sites during the wet season using the 100 mL sampling compared to the 15 mL sampling (Fig. 4A).

In real terms, when one is comparing positive detection or not, the 100 mL sampling method gave *L. lorica* detections at five of the seven possible sites, whereas the 15 mL method gave detections at three of the seven sites (Table 2). Additionally, the 100 mL sampling method gave *L. lorica* detections at both sites where only about 100 adults are present directly upstream (*i.e*., sites 2 and 6), whereas no *L. lorica* detections were made at these sites using the 15 mL method (Table 2). Finally, the 100 mL sampling method gave 12.5% *L. lorica* detections across replicates at the furthest site (site 10; 22.8 km downstream); whereas the 15 mL method gave no *L. lorica* detections at this site.

Comparison of *L. nannotis* detection from precipitation of 100 mL samples *vs*. 15 mL samples was not possible due to a laboratory issue during screening some of the 100 mL water samples, detailed above. These sites are shown as no data in Table 2 (dashes) and Fig. 1B (0 symbols). The only comparison that can be made is the observation that the detection frequency for *L. nannotis* at sites 6–8 was markedly higher for the 100 mL sampling method than the 15 mL method (Table 2). Using the 15 mL sampling method, *L. nannotis* was detected at all but one site (site 9; 17.1 km downstream) but detection frequencies were low (<10% at six out of the nine sites with detections; Table 2).

### Was the more abundant species more readily detected?

We assumed that *L. nannotis* would be more readily detected than *L. lorica* at sites they could both be detected because there is estimated to be at least an order of magnitude more *L. nannotis* than *L. lorica* upstream of all these sites (C. Hoskin, 2019, unpublished data; Table S5). This was reflected in the percentage of positive eDNA detections across sites using the 15 mL methods, with *L. nannotis* detections being noticeably higher (Table 2). Further, when using the 15 mL samples, sites 2, 5, 6 and 10 did not show positive *L. lorica* eDNA detections, but all of these sites had positive detections of *L. nannotis* eDNA (43.3%, 3.3%, 25%, 5%, respectively). A Wilcoxon signed-rank test, using the paired average probabilities for each species at the seven sites where both species are present upstream (*i.e*., sites 2, 4–7, 9, 10) (Table 2; Fig. 1), revealed that *L. nannotis* was indeed more readily detected (mean difference 16.05, z = −1.89, W = 1.5, *P* < 0.05). Site occupancy modelling also revealed a higher probability of detecting *L. nannotis* than *L. lorica* eDNA at sites up to 4 km downstream from the source population (Fig. 4B). At sites located 10 km downstream of the source populations and beyond, the eDNA detection probability for both species was very similar (Fig. 4B).

In real terms, when one is comparing positive detection or not, *L. nannotis* was detected using the 15 mL sampling method at six of the seven possible sites, whereas *L. lorica* was only detected at three of the seven sites (Table 2). A similar comparison could not be tested for the 100 mL sampling method due to the mechanical failure of the qPCR machine during the *L. nannotis* screening for most sites (detailed above). For the 100 mL samples, only two sites could be compared (sites 6 and 7), and the percentage of positive eDNA detections was higher for *L. nannotis* at both of these (Table 2).

### Was detection probability higher in the wet season than the dry season?

The primary objective of conducting eDNA sampling during both the wet and dry season was to determine whether far-downstream eDNA detection was possible under both high stream flow (wet season) and low stream flow (dry season). Dry season sampling just focussed on key subset of the sites. Importantly, both *L. lorica* and *L. nannotis* were also detected at most far-downstream sites during the dry season sampling (Table S4). In the dry season sampling, the percentage of positive *L. lorica* eDNA detections at the main population (site 6) was 6.7% and 1.7% at both of the two furthest downstream sites (17.1 and 22.8 km); but there were no detections at site 7 (10 km) (Table S4). *Litoria nannotis* percentage of positive eDNA detections in the dry season sampling was 3.3% at site 7 (10 km) and 1.7% at sites 9 and 10 (17.1 and 22.8 km downstream, respectively); but there were no detections at site 8 (Table S4).

*Litoria lorica* detection frequencies were higher during the wet season (high flow) sampling than the dry season (low flow) sampling at all four sites they could have been detected at (sites 6, 7, 9, 10; Fig. 3; Table S4). The site occupancy models showed slightly higher probability of *L. lorica* eDNA detection during the wet season than during the dry season (Fig. 4C). Statistical testing using a Wilcoxon signed-rank test was not possible due to too few site comparisons. Comparison of *L. nannotis* wet *vs*. dry season sampling was not possible due to the laboratory issue outlined above (Table S4).

## Discussion

Globally, hundreds of amphibian species are listed as Critically Endangered or ‘missing’ (IUCN, 2020; Stuart et al., 2004). Conservation of these species is dependent on finding remnant populations (Gillespie et al., 2020), which is a challenge in remote, rugged environments using traditional survey techniques. Sampling water in downstream sections of catchments and screening for eDNA of target species could be a valuable tool for surveying for endangered and ‘missing’ frog populations in these environments. However, the key question is whether small amphibian populations can be detected kilometres downstream.

Our study demonstrates far-downstream detection of an Endangered and a Critically Endangered frog species, with fairly consistent detection at sites up to 22.8 km downstream. These represent considerably further downstream eDNA detection distances than most previous studies. Cage experiments (typically involving very small numbers of individuals) have generally had detections limited to hundreds of meters (Table S1), but out to five kilometers for a larger biomass of caged fish (Laporte et al., 2020). Research on wild populations has also shown maximum eDNA detection distances in the order of hundreds of meters to five kilometers (*e.g*., Civade et al., 2016; Jane et al., 2015; Stoeckle, Kuehn & Geist, 2016; Wilcox et al., 2016; Wacker et al., 2019). The few previous studies that have found ‘far-downstream’ detection (*i.e*., >10 km) have been for species that were either abundant at the source (Deiner & Altermatt, 2014; Itakura et al., 2019; Pont et al., 2018) or species for which the downstream limit was not accurately known (Deiner & Altermatt, 2014; Pont et al., 2018). Importantly, downstream limits are well-resolved for our target species in this catchment. This is due to ecology and thorough field surveys. Both species are restricted to waterfalls and cascades (all life stages) and these habitat features end abruptly on these streams due to particular stream topography in the region. These downstream limits have been surveyed in detail throughout this catchment, using traditional techniques. We are therefore confident that our far downstream eDNA detection distances are not confounded by overlooked populations in between.

Our results showed the expected pattern of higher detection of the more abundant species (for the 15 mL sampling, which was the only method with enough comparisons; Fig. 4B). *Litoria lorica* and *L. nannotis* are at similar abundance in the two stream sections that they co-occur but *L. nannotis* also occurs in many other areas of the catchment (Fig. 1), and it is estimated that there is an order of magnitude more *L. nannotis* upstream from all of our sampling sites (Table S5). A study testing eDNA detection downstream from sites containing different densities of eastern hellbender salamanders also found higher detection success downstream from larger populations (Olson, Briggler & Williams, 2012). More generally, a positive correlation has been found between eDNA concentration and population abundance in both field-based studies and mesocosm experiments for amphibians, fish and invertebrates (Yates, Fraser & Derry, 2019).

*Litoria lorica* was of particular importance in our study because the catchment contains the only two populations of this species, globally. The size of both these populations is regularly estimated through detailed field surveys and monitoring, with the larger ‘main’ population being about 1,000 adults and the smaller ‘reintroduced’ population being about 100 adults at the time of our study (C. Hoskin, 2019, unpublished data). These population sizes are particularly relevant to our main aim of resolving how far downstream small, remnant frog populations can be detected. For the larger population, the 15 mL sampling method gave poor detection success, but the 100 mL method and water filtration gave consistent detection at the far-downstream sites (Fig. 1), including high detection frequencies at some sites (Table 2). In contrast, detection success of approximately 100 adults was very low. The 100 mL sampling method gave positive detections of *L. lorica* at sites immediately downstream of 100 adults (*i.e*., sites 2 and 6) but the 15 mL method did not (Table 2). Detection success further downstream of the reintroduced population (*i.e*., sites 4 and 5) was very low (Table 2); even filtration at site 5 (2.7 km downstream) revealed no detections.

Far-downstream detection of the main *L. lorica* population, and *L. nannotis*, was achieved both during high stream flow conditions of the wet season and during low stream flow conditions of the dry season. Detection frequencies and median probability of detection were higher in the wet season sampling (Figs. 3 and 4C; Table S4). Seasonal variations in eDNA detection could be due to changes in the species’ habitat use or ecology (Goldberg et al., 2011; Spear et al., 2015). This is unlikely the cause of differences in our system because (i) *L. lorica* and *L. nannotis* inhabit cascades/waterfalls all year and they do not move away from these discrete sections of suitable habitat (Puschendorf et al., 2011), and (ii) based on knowledge of the species’ breeding (C. Hoskin, 2019, unpublished data), there were probably many tadpoles in the stream at the time of the dry season sampling (late October) and few at the time of the wet season sampling (early April); yet, detection was higher during the wet season. We therefore hypothesize that the apparent seasonal differences in eDNA detectability are due to a higher water discharge during the wet season, with higher water discharge typically resulting in higher eDNA detectability downstream (*e.g*., Jane et al., 2015; Jerde et al., 2016; Robinson et al., 2019; Shogren et al., 2017, Shogren et al., 2018). Regardless of possible seasonal differences, the important result in our study was far-downstream detection (to 22.8 km) under both stream flow conditions.

We used three eDNA sampling methods during the wet season sampling at the far-downstream sites. The comparisons across these four sites are too few to statistically test detection frequency across all three methods. However, detection frequency is noticeably higher for the filtration method than for the two water sampling methods (Table 2). This is not surprising given that the amount of water screened per site was about three orders of magnitude larger for filtration than for the 15 and 100 mL methods (Table 2; Table S2). Our filtered volumes were also about two orders of magnitude larger than the commonly used sample volumes for eDNA studies (reviewed by Rees et al., 2017), which could be the reason behind our much further downstream distance detection than previous filtering studies involving small populations (*e.g*., Stoeckle, Kuehn & Geist, 2016; Table S1).

Water filtration is the most widely used method for eDNA sampling because it can screen large volumes of water and hence maximize the probability of capturing eDNA (Eichmiller, Bajer & Sorensen, 2014; Pilliod et al., 2013; Sepulveda et al., 2019). However, filtration of large volumes is time-consuming and requires expensive gear that is not readily available (but see Laramie et al., 2015 for examples of on-site filtration). Additionally, large volume filtration units are typically bulky, which is not practical for carrying to remote sites. There is also the potential risk of contamination in the field because the filtering gear may not be possible to completely decontaminate, and carrying multiple set of single-use equipment to remote sites would be challenging (Huerlimann et al., 2020). In contrast, using new, clean plastic tubes or bottles to collect water (for subsequent direct precipitation) is practical for remote sites, and limits the risk of cross-contamination in the field.

We used two water collection/precipitation methods: precipitation of 15 mL and 100 mL stream water samples. The detection frequencies, and probabilities determined by the site occupancy models, were higher for the 100 mL sampling method than the 15 mL method. An important part of this is that the 100 mL sampling method gave 37.5%, 16.7% and 12.5% positive detections across replicates at all three far-downstream sites that *L. lorica* could have been detected at (*i.e*., at 10 km, 17.1 km and 22.8 km downstream, respectively), whereas the 15 mL method gave just 3.3%, 3.3% and 0 positive detections. Additionally, the 100 mL sampling method detected *L. lorica* at both sites that are immediately downstream of about 100 adults a (*i.e*., sites 2 and 6), whereas the 15 mL method did not detect *L. lorica* at either. We conclude that the 100 mL water sampling was significantly more reliable for detecting *L. lorica* than the15 mL sampling. The 100 mL samples are bulkier and heavier for remote sampling on foot, but not prohibitively so, and processing any volume larger than 100 mL becomes less practical in the laboratory (due to capacity of standard centrifuges).

Direct water precipitation has rarely been used for detection of stream-dwelling organisms because it is assumed that large volumes of water need to be filtered, particularly if the aim is far-downstream detection (Eichmiller, Bajer & Sorensen, 2014; Pilliod et al., 2013; Sepulveda et al., 2019). In contrast, direct water collection and subsequent precipitation has been used for detection of rare aquatic species in ponds and wetlands (*e.g*., Dejean et al., 2012; Ficetola et al., 2008; Rees et al., 2017), and some studies have found the method to be equally or more effective than filtration. This is potentially because water precipitation allows for capturing extracellular eDNA that is mostly lost during filtration (DeFlaun, Paul & Davis, 1986; Liang & Keeley, 2013). For example, Raemy & Ursenbacher (2018) did not find significant differences between filtration and precipitation for detection of a freshwater turtle in natural ponds. Similarly, Piaggio et al. (2014) found that precipitating 15 mL of water from an experimental pen and purifying it using the QIAamp DNA Micro Kit was slightly more efficient at detecting the target species than filtering 2 L of water through a 0.75 µm glass fibre filter. While we acknowledge the fact that filtration of large water volumes is ideal for detection of rare species, our study is novel in showing that direct water precipitation of small volumes can also be an effective method for detecting rare stream-dwelling species many kilometres downstream.

It is possible that environmental characteristics of our stream system were conducive for far-downstream eDNA detection; however, this could not be assessed in our study because we did not measure factors such as water temperature, pH or nutrient load. We hypothesize that a combination of low eDNA degradation and high resuspension in our system may have contributed to our particularly long detection distances. Stream water in the Wet Tropics mountains, including in this catchment, is very clear and of low nutrient load (Brodie & Mitchell, 2006), and hence reduced bacterial action. Nutrient-rich systems with high bacterial load have been shown to have rapid eDNA degradation (Eichmiller, Best & Sorensen, 2016). Additionally, the consistently shallow nature of the streams in our catchment, along with perennial flow, likely generates high/continuous eDNA resuspension. This will especially be the case during the wet season, when high stream flow events following heavy rain can resuspend eDNA and transport it downstream (Shogren et al., 2017).

### Implications for finding populations of threatened and ‘missing’ amphibian species

Our study shows that rare aquatic species can be detected over 20 km downstream, including a Critically Endangered frog population of about 1,000 adults. This is a significant advance on previous studies of small population detection using eDNA, which have generally demonstrated detection distances of hundreds of meters rather than kilometres (Olson, Briggler & Williams, 2012; Stoeckle, Kuehn & Geist, 2016; Wacker et al., 2019). Further, our study shows that reliable far-downstream eDNA detection is not only possible using filtration of large volumes but also by precipitating water volumes of 100 mL. In contrast, smaller water volumes of 15 mL were not reliable for far-downstream detection of these frogs. The 100 mL sampling method was also successful for detecting a very small population of about 100 individuals of *L. lorica* immediately downstream. None of the three methods were successful for detecting the very small population kilometres downstream, suggesting there may be a population size threshold for long distance eDNA detection using the methods and replicate numbers we used. Further studies should be conducted on other taxa, either using wild populations or caged individuals, to determine population size thresholds for far-downstream eDNA detection.

Our results have applicability to the hundreds of Critically Endangered and ‘missing’ stream frogs, globally, many of which are/were found in remote mountainous areas (Scheele et al., 2019). We suggest the following methodology. First, a water filtration method is recommended if a stream site is accessible (*e.g*., where there is a road crossing), and we recommend filtering large volumes of stream water (>1,000 L) to detect rare species. Second, the method that best balances reliability and practicality is collecting moderate water volumes (*e.g*., individual 100 mL samples, or 500 mL and then precipitating eDNA from 100 mL subsamples). Collecting water samples does not require training or special equipment and many bottles can be carried in a backpack. Third, although we could not test the effect of stream flow statistically, the data suggests that water collection during higher flow periods (*e.g*., the wet season in our system) gives higher detection than during low flow periods. However, we predict lower detection during flooding conditions due to the dilution of eDNA in massive water volumes. Fourth, we suggest screening in readily accessible downstream areas and then working upstream from there if: (i) a positive detection is made, or (ii) there is strong reason to believe a very small population remains undetected upstream (*e.g*., the last known historic site or an unconfirmed recent sighting).

In our system, the next step will be screening downstream sites in all catchments where *L. lorica* was known to occur before disease-induced declines, or likely occurred but was undetected prior to declines. The only two known populations of *L. lorica* occur in the catchment we studied herein, on the western Carbine Tableland, but the species was previously known from a major catchment on the eastern Carbine Tableland, and several streams in the Thornton Peak uplands (Cunningham, 2002). The downstream detection distances found in the present study suggest that we can use filtration or the 100 mL sampling method to sample accessible downstream areas of all major catchments flowing off these two upland areas to fairly reliably search for ‘missing’ populations across the entire historic distribution.

More broadly, a key remaining question is what positive eDNA detections say about abundance and distance from the population. This could be further tested in either of these species, by incorporating more sites, more repeat sampling across seasons, and additional data on environmental factors, such as flow rate and volume. Regardless, the simple approach we present in the present study could be used to try find the remaining populations of hundreds of other amphibians, globally, that are so urgently required to facilitate management.

## Supplemental Information

10.7717/peerj.12013/supp-1 Supplemental Information 1 Supplemental Tables and Figures cited in the article. Tables S1–S7 and Figs. S1 and S2 providing supporting documentation to the article.

10.7717/peerj.12013/supp-2 Supplemental Information 2 Detection data for eDNA occupancy models. Data was used to test the probability of detection of (a) *L. lorica* using the 15 and 100 mL samples; (b) *L. lorica* during wet and dry seasons using the 100 mL samples; and (c) *L. lorica* and *L. nannotis* during wet season using the 15 mL samples.

10.7717/peerj.12013/supp-3 Supplemental Information 3 Survey data for eDNA occupancy models. Data was used to test the probability of detection of (a) *L. lorica* using the 15 and 100 mL samples; (b) *L. lorica* during wet and dry seasons using the 100 mL samples; and (c) *L. lorica* and *L. nannotis* during wet season using the 15 mL samples. Upstream population abundances are estimated from traditional field surveys (C. Hoskin, unpublished data).

10.7717/peerj.12013/supp-4 Supplemental Information 4 *Litoria lorica* and *L. nannotis* sequences. Amplicon sequences obtained from the target species eDNA.

## Additional Information and Declarations

### Field Study Permissions

The following information was supplied relating to field study approvals (*i.e*., approving body and any reference numbers): The Queensland Government approved this study (Permit WITK18662017).

### DNA Deposition

The following information was supplied regarding the deposition of DNA sequences: *Litoria lorica* and *L. nannotis* sequences are available in GenBank: MZ330818 and MZ330819 (*L. lorica*) and MZ383982 and MZ383983 (*L. nannotis*).

### Data Availability

The following information was supplied regarding data availability: Raw data are available in the Supplemental Materials.

## References

[ref-1] Anstis M (2013). Tadpoles and frogs of Australia.

[ref-2] Barnes MA, Turner CR, Turner CR (2016). The ecology of environmental DNA and implications for conservation genetics. Conservation Genetics.

[ref-3] Bedwell ME, Goldberg CS (2020). Spatial and temporal patterns of environmental DNA detection to inform sampling protocols in lentic and lotic systems. Ecology and Evolution.

[ref-4] Brodie J, Mitchell A (2006). Sediments and nutrients in north Queensland tropical streams: changes with agricultural development and pristine condition status.

[ref-5] Bustin SA, Benes V, Garson JA, Hellemans J, Huggett J, Kubista M, Mueller R, Nolan T, Pfaffl MW, Shipley GL, Vandesompele J, Wittwer CT (2009). The MIQE guidelines: minimum information for publication of quantitative real-time PCR experiments. Clinical Chemistry.

[ref-6] Calter PA, Calter MA (2011). Technical mathematics with calculus.

[ref-7] Cao Y, Griffith JF, Dorevitch S, Weisberg SB (2012). Effectiveness of qPCR permutations, internal controls and dilution as means for minimizing the impact of inhibition while measuring Enterococcus in environmental waters. Journal of Applied Microbiology.

[ref-8] Civade R, Dejean T, Valentini A, Roset N, Raymond JC, Bonin A, Pont D, Taberlet P, Pont D, Garcia de Leaniz C (2016). Spatial representativeness of environmental DNA metabarcoding signal for fish biodiversity assessment in a natural freshwater system. PLOS ONE.

[ref-9] Cunningham M (2002). Identification and evolution of Australian Torrent Treefrogs (Anura: Hylidae: *Litoria nannotis* group). Memoirs of the Queensland Museum.

[ref-10] DeFlaun MF, Paul JH, Davis D (1986). Simplified method for dissolved DNA determination in aquatic environments. Applied and Environmental Microbiology.

[ref-11] Deiner K, Altermatt F (2014). Transport distance of invertebrate environmental DNA in a natural river. PLOS ONE.

[ref-12] Deiner K, Fronhofer EA, Mächler E, Walser JC, Altermatt F (2016). Environmental DNA reveals that rivers are conveyer belts of biodiversity information. Nature Communications.

[ref-13] Dejean T, Valentini A, Miquel C, Taberlet P, Bellemain E, Miaud C (2012). Improved detection of an alien invasive species through environmental DNA barcoding: the example of the American bullfrog *Lithobates catesbeianus*. Journal of Applied Ecology.

[ref-14] Dorazio RM, Erickson RA (2018). Ednaoccupancy: an R package for multiscale occupancy modelling of environmental DNA data. Molecular Ecology Resources.

[ref-15] Edmunds RC, Burrows D (2020). Got Glycogen?: Development and multispecies validation of the novel preserve, precipitate, lyse, precipitate, purify (PPLPP) workflow for environmental DNA extraction from Longmire’s preserved water samples. Journal of Biomolecular Techniques.

[ref-16] Edmunds RC, Villacorta-Rath C, Huerlimann R, Burrows DW (2019). Development of eDNA assays for monitoring three endangered frog species (Litoria dayi, L. lorica and L. nannotis) in Australia’s wet tropics.

[ref-17] Eichmiller JJ, Bajer PG, Sorensen PW (2014). The relationship between the distribution of common Carp and their environmental DNA in a small lake. PLOS ONE.

[ref-18] Eichmiller JJ, Best SE, Sorensen PW (2016). Effects of temperature and trophic state on degradation of environmental DNA in lake water. Environmental Science and Technology.

[ref-19] Feng X, Porporato A, Rodriguez-Iturbe I (2013). Changes in rainfall seasonality in the tropics. Nature Climate Change.

[ref-20] Ficetola GF, Manenti R, Taberlet P (2019). Environmental DNA and metabarcoding for the study of amphibians and reptiles: species distribution, the microbiome, and much more. Amphibia Reptilia.

[ref-21] Ficetola GF, Miaud C, Pompanon F, Taberlet P (2008). Species detection using environmental DNA from water samples. Biology Letters.

[ref-22] Furlan EM, Gleeson D, Hardy CM, Duncan RP (2016). A framework for estimating the sensitivity of eDNA surveys. Molecular Ecology Resources.

[ref-23] Gillespie GR, Roberts JD, Hunter D, Hoskin CJ, Alford RA, Heard GW, Hines H, Lemckert F, Newell D, Scheele BC (2020). Status and priority conservation actions for Australian frog species. Biological Conservation.

[ref-24] Goldberg CS, Pilliod DS, Arkle RS, Waits LP (2011). Molecular detection of vertebrates in stream water: a demonstration using Rocky Mountain tailed frogs and Idaho giant salamanders. PLOS ONE.

[ref-25] Hoskin CJ, Puschendorf R (2014). The importance of peripheral areas for biodiversity conservation: with particular focus on endangered rainforest frogs of the Wet Tropics and Eungella.

[ref-26] Huerlimann R, Cooper MK, Edmunds RC, Villacorta-Rath C, Le Port A, Robson HLA, Strugnell JM, Burrows D, Jerry DR (2020). Enhancing tropical conservation and ecology research with aquatic environmental DNA methods: an introduction for non-environmental DNA specialists. Animal Conservation.

[ref-27] Itakura H, Wakiya R, Yamamoto S, Kaifu K, Sato T, Minamoto T (2019). Environmental DNA analysis reveals the spatial distribution, abundance, and biomass of Japanese eels at the river-basin scale. Aquatic Conservation: Marine and Freshwater Ecosystems.

[ref-28] IUCN (2020). The IUCN red list of threatened species. Version 2020-1. https://www.iucnredlist.org.

[ref-29] Jane SF, Wilcox TM, Mckelvey KS, Young MK, Schwartz MK, Lowe WH, Letcher BH, Whiteley AR (2015). Distance, flow and PCR inhibition: eDNA dynamics in two headwater streams. Molecular Ecology Resources.

[ref-30] Jerde CL, Olds BP, Shogren AJ, Andruszkiewicz EA, Mahon AR, Bolster D, Tank JL (2016). Influence of stream bottom substrate on retention and transport of vertebrate environmental DNA. Environmental Science and Technology.

[ref-31] Klymus KE, Merkes CM, Allison MJ, Goldberg CS, Helbing CC, Hunter ME, Jackson CA, Lance RF, Mangan AM, Monroe EM, Piaggio AJ, Stokdyk JP, Wilson CC, Richter CA (2019). Reporting the limits of detection and quantification for environmental DNA assays. Environmental DNA.

[ref-32] Laporte M, Bougas B, Côté G, Champoux O, Paradis Y, Morin J, Bernatchez L (2020). Caged fish experiment and hydrodynamic bidimensional modeling highlight the importance to consider 2D dispersion in fluvial environmental DNA studies. Environmental DNA.

[ref-33] Laramie MB, Pilliod DS, Goldberg CS, Strickler KM (2015). Environmental DNA sampling protocol—filtering water to capture DNA from aquatic organisms: U.S. Geological Survey techniques and methods. https://pubs.usgs.gov/tm/02/a13/tm2a13.pdf.

[ref-34] Laurance WF, McDonald KR, Speare R (1996). Epidemic disease and the catastrophic decline of Australian rain forest frogs. Conservation Biology.

[ref-35] Lewis S, Bainbridge Z, Stevens T, Garzon-Garcia A, Chen C, Burton J, Bahadori M, Rezaei Rashti M, Gorman J, Smithers S, Olley J, Moody P, Dehayr R (2018). Sediment tracing from the catchment to reef 2016 to 2018 : flood plume, marine sediment trap and logger data time series. http://www.nesptropical.edu.au/index.php/final-reports-round-2/.

[ref-36] Liang Z, Keeley A (2013). Filtration recovery of extracellular DNA from environmental water samples. Environmental Science and Technology.

[ref-37] Longmire JL, Maltbie M, Baker RJ (1997). Use of “lysis buffer” in DNA isolation and its implication for museum collections.

[ref-38] Lopes CM, Id O, Valentini A, Id O, Lyra CIO, Id O, Issue S (2020). Lost and found: frogs in a biodiversity hotspot rediscovered with environmental DNA. Molecular Ecology.

[ref-39] Lough JM (1993). Variations of some seasonal rainfall characteristics in Queensland, Australia: 1921–1987. International Journal of Climatology.

[ref-40] Meyer EA, Hines HB, Clarke JM, Hoskin CJ (2020). An update on the status of wet forest stream-dwelling frogs of the Eungella region. Proceedings of the Royal Society of Queensland.

[ref-41] Olson ZH, Briggler JT, Williams RN (2012). An eDNA approach to detect eastern hellbenders (*Cryptobranchus alleganiensis*) using samples of water. Wildlife Research.

[ref-42] Piaggio AJ, Engeman RM, Hopken MW, Humphrey JS, Keacher KL, Bruce WE, Avery ML (2014). Detecting an elusive invasive species: a diagnostic PCR to detect Burmese python in Florida waters and an assessment of persistence of environmental DNA. Molecular Ecology Resources.

[ref-43] Pilliod DS, Goldberg CS, Arkle RS, Waits LP (2013). Estimating occupancy and abundance of stream amphibians using environmental DNA from filtered water samples. Canadian Journal of Fisheries and Aquatic Sciences.

[ref-44] Pilliod DS, Goldberg CS, Arkle RS, Waits LP (2014). Factors influencing detection of eDNA from a stream-dwelling amphibian. Molecular Ecology Resources.

[ref-45] Pont D, Rocle M, Valentini A, Civade Rël, Jean P, Maire A, Roset N, Schabuss M, Zornig H, Dejean T (2018). Environmental DNA reveals quantitative patterns of fish biodiversity in large rivers despite its downstream transportation. Science of the Total Environment.

[ref-46] Puschendorf R, Hoskin CJ, Cashins SD, McDonald K, Skerratt LF, Vanderwal J, Alford RA (2011). Environmental refuge from disease-driven amphibian extinction. Conservation Biology.

[ref-47] Raemy M, Ursenbacher S (2018). Detection of the European pond turtle (*Emys orbicularis*) by environmental DNA: is eDNA adequate for reptiles?. Amphibia Reptilia.

[ref-48] Rees HC, Baker CA, Gardner DS, Maddison BC, Gough KC (2017). The detection of great crested newts year round via environmental DNA analysis. BMC Research Notes.

[ref-49] Rees HC, Maddison BC, Middleditch DJ, Patmore JRM, Gough KC (2014). The detection of aquatic animal species using environmental DNA—a review of eDNA as a survey tool in ecology. Journal of Applied Ecology.

[ref-50] Robinson AT, Paroz YM, Clement MJ, Franklin TW, Dysthe JC, Young MK, McKelvey KS, Carim KJ (2019). Environmental DNA sampling of small-bodied minnows: performance relative to location, species, and traditional sampling. North American Journal of Fisheries Management.

[ref-51] Santas AJ, Persaud T, Wolfe BA, Bauman JM (2013). Noninvasive method for a statewide survey of eastern hellbenders *Cryptobranchus alleganiensis* using environmental DNA. International Journal of Zoology.

[ref-52] Sasso T, Lopes CM, Valentini A, Dejean T, Zamudio KR, Haddad CFB, Martins M (2017). Environmental DNA characterization of amphibian communities in the Brazilian Atlantic forest: potential application for conservation of a rich and threatened fauna. Biological Conservation.

[ref-53] Scheele BC, Gillespie GR (2018). The extent and adequacy of monitoring for Australian threatened frog species. Monitoring Threatened Species and Ecological Communities.

[ref-54] Scheele BC, Pasmans F, Skerratt LF, Berger L, Martel A, Beukema W, Acevedo AA, Burrowes PA, Carvalho T, Catenazzi A, De la Riva I, Fisher MC, Flechas SV, Foster CN, Frías-Álvarez P, Garner TWJ, Gratwicke B, Guayasamin JM, Hirschfeld M, Kolby JE, Kosch TA, La Marca E, Lindenmayer DB, Lips KR, Longo AV, Maneyro Rúl, McDonald CA, Mendelson J, Palacios-Rodriguez P, Parra-Olea G, Richards-Zawacki CL, Rödel M-O, Rovito SM, Soto-Azat C, Toledo Lís F, Voyles J, Weldon Cé, Whitfield SM, Wilkinson M, Zamudio KR, Canessa S (2019). Amphibian fungal panzootic causes catastrophic and ongoing loss of biodiversity. Science.

[ref-55] Scheele BC, Skerratt LF, Grogan LF, Hunter DA, Clemann N, McFadden M, Newell D, Hoskin CJ, Gillespie GR, Heard GW, Brannelly L, Roberts AA, Berger L (2017). After the epidemic: ongoing declines, stabilizations and recoveries in amphibians afflicted by chytridiomycosis. Biological Conservation.

[ref-56] Schumer G, Crowley K, Maltz E, Johnston M, Anders P, Blankenship S (2019). Utilizing environmental DNA for fish eradication effectiveness monitoring in streams. Biological Invasions.

[ref-57] Sepulveda AJ, Schabacker J, Smith S, Al-Chokhachy R, Luikart G, Amish SJ (2019). Improved detection of rare, endangered and invasive trout in using a new large-volume sampling method for eDNA capture. Environmental DNA.

[ref-58] Shogren AJ, Tank JL, Andruszkiewicz E, Olds B, Mahon AR, Jerde CL, Bolster D (2017). Controls on eDNA movement in streams: transport, retention, and resuspension. Scientific Reports.

[ref-59] Shogren AJ, Tank JL, Egan SP, August O, Rosi EJ, Hanrahan BR, Renshaw MA, Gantz CA, Bolster D (2018). Water flow and biofilm cover influence environmental DNA detection in recirculating streams. Environmental Science and Technology.

[ref-60] Spear SF, Groves JD, Williams LA, Waits LP (2015). Using environmental DNA methods to improve detectability in a hellbender (*Cryptobranchus alleganiensis*) monitoring program. Biological Conservation.

[ref-61] Stevens T (2020). Patent No. WO/2020/132718. World Intellectual Property Organization, International Bureau. https://www.wipo.int/patentscope/en/.

[ref-62] Stoeckle BC, Kuehn R, Geist J (2016). Environmental DNA as a monitoring tool for the endangered freshwater pearl mussel (*Margaritifera margaritifera* L.): a substitute for classical monitoring approaches?. Aquatic Conservation: Marine and Freshwater Ecosystems.

[ref-63] Stuart SN, Chanson JS, Cox NA, Young BE, Rodrigues ASL, Fischman DL, Waller RW (2004). Status and trends of amphibian declines and extinctions worldwide. Science.

[ref-64] Villacorta-Rath C, Adekunle A, Edmunds RC, Strugnell JM, Schwarzkopf L, Burrows D (2020). Can environmental DNA be used to detect first arrivals of the cane toad, *Rhinella marina*, into novel locations?. Environmental DNA.

[ref-65] Wacker S, Fossøy F, Larsen BM, Brandsegg H, Sivertsgård R, Karlsson S (2019). Downstream transport and seasonal variation in freshwater pearl mussel (*Margaritifera margaritifera*) eDNA concentration. Environmental DNA.

[ref-66] Wilcox TM, McKelvey KS, Young MK, Sepulveda AJ, Shepard BB, Jane SF, Whiteley AR, Lowe WH, Schwartz MK (2016). Understanding environmental DNA detection probabilities: a case study using a stream-dwelling char *Salvelinus fontinalis*. Biological Conservation.

[ref-67] Yates MC, Fraser DJ, Derry AM (2019). Meta-analysis supports further refinement of eDNA for monitoring aquatic species-specific abundance in nature. Environmental DNA.

