# Supplementary material for: Long distance (>20 km) downstream detection of endangered stream frogs suggests an important role for eDNA in surveying for remnant amphibian populations
Source: PeerJ. 2021 Sep 27;9:e12013. doi: 10.7717/peerj.12013 (PMC8483009; doi:10.7717/peerj.12013)

**TABLE S1.** Cage-experiment studies testing eDNA downstream transport in riverine systems. All studies involved filtration (F) of water samples.

| **Reference** | **Study species** | **Study system(s)** | **# cages used** | **Caged population size or biomass** | **Time to sample collection** | **Collection method and sample volume** | **Downstream distance tested (m)** | **Maximum downstream distance detection (m)** |
| --- | --- | --- | --- | --- | --- | --- | --- | --- |
| Curtis and Larson (2020) | Red swamp crayfish (*Procambarus clarkii*) carcasses | 1 stream | 5 | 3 crayfish carcasses/cage | 1-28 days | (F) 1.0 µm cellulose nitrate filters; 5 x 250 mL replicates at 0 m from cages and 4 x 250 mL replicates at 20 m from cages | 0 and 20 | No amplification |
| Gasparini *et al.* (2020) | Wavy-rayed lampmussel (*Lampsilis fasciola*) | 2 rivers | 1/river | 1 mussel/cage; 10 mussels/cage | 24 and 48 h | (F) 5 μm nitrocellulose filter cartridges; 2 × 1 L replicates | 0, 10, 50 and 100 | 0 |
| Jane *et al.* (2015) | Brook trout (*Salvelinus fontinalis*) | 2 streams | 1/stream | 5 fish/cage | 24 h | (F) 1.5 μm glass fibre filter; 1 x 6 L replicate | Nine sites between 27.5 - 239.5 | 239.5 |
| Laporte *et al.* (2020) | Brown trout (*Salmo trutta*) | 2 water masses flowing in parallel | 1/water mass | 49 and 50 fish/cage | 48 and 72 h | (F) 1.2 μm glass microfiber filter; 3-5 x 250 mL replicates/water mass/distance | 10, 100, 500, 1000 and 5000 | 5000 |

**TABLE S1.** continued

| **Reference** | **Study species** | **Study system(s)** | **# cages used** | **Caged population size or biomass** | **Time to sample collection** | **Collection method and sample volume** | **Downstream distance tested (m)** | **Maximum downstream distance detection (m)** |
| --- | --- | --- | --- | --- | --- | --- | --- | --- |
| Robinson *et al.* (2019) | Spikedace (*Meda fulgida*) and Loach Minnow (*Rhinichthys cobitis*) | 2 streams | 1/species/stream | 20 Spikedace; 15 Loach Minnows | intervals from 2 h | (F) 1.5 μm glass-fiber filter; 3 x 5 L replicates/distance | 0, 50, 100, 200, 300, 400 and 500 | 500 |
| Pilliod *et al.* (2014) | Idaho giant salamander (*Dicamptodon aterrimus*) | 2 streams | 1 and 5 | 1 salamander/cage | 1, 3, 6, 12 and 24 h | (F) 0.45 μm cellulose nitrate filter; 1 x 2 L replicate | 5 and 50 | 5 |
| Schumer *et al.* (2019) | Rainbow trout (*Oncorhynchus mykiss*) and Smallmouth bass (*Micropterus dolomieu*) carcass | 3 streams | 1/stream | 1 fish/cage | 16-24 h | (F) 0.45 μm Sterivex™ filter; 1–9 x 0.5–8 L replicates | 100, 250, 500 and 1000 | 500 |
| Wood *et al.* (2020) | Atlantic salmon (*Salmo salar*) | 1 stream | 1 | 1, 4, 8, and 20 fish | 24-48 h | (F) 1.5 μm glass-fibre filter; 3 reps of 1 L | 10, 100, 500, and 1000 | 1000 |

**References**

Curtis, A. N. and Larson, E. R. (2020) ‘No evidence that crayfish carcasses produce detectable environmental DNA (eDNA) in a stream enclosure experiment’, *PeerJ*, 8, p. e9333. doi: 10.7717/peerj.9333.

Gasparini, L. *et al.* (2020) ‘Detection of freshwater mussels (Unionidae) using environmental DNA in riverine systems’, *Environmental DNA*, (July 2019), pp. 1–9. doi: 10.1002/edn3.71.

Jane, S. F. *et al.* (2015) ‘Distance, flow and PCR inhibition: eDNA dynamics in two headwater streams’, *Molecular Ecology Resources*, 15(1), pp. 216–227. doi: 10.1111/1755-0998.12285.

Laporte, M. *et al.* (2020) ‘Caged fish experiment and hydrodynamic bidimensional modeling highlight the importance to consider 2D dispersion in fluvial environmental DNA studies’, *Environmental DNA*, (April), pp. 1–11. doi: 10.1002/edn3.88.

Pilliod, D. S. *et al.* (2014) ‘Factors influencing detection of eDNA from a stream-dwelling amphibian’, *Molecular Ecology Resources*, 14(1), pp. 109–116. doi: 10.1111/1755-0998.12159.

Robinson, A. T. *et al.* (2019) ‘Environmental DNA sampling of small-bodied minnows: Performance relative to location, species, and traditional sampling’, *North American Journal of Fisheries Management*, 39(5), pp. 1073–1085. doi: 10.1002/nafm.10344.

Schumer, G. *et al.* (2019) ‘Utilizing environmental DNA for fish eradication effectiveness monitoring in streams’, *Biological Invasions*. Springer International Publishing, 21(11), pp. 3415–3426. doi: 10.1007/s10530-019-02056-z.

Wood, Z. T. *et al.* (2020) ‘Experimental assessment of optimal lotic eDNA sampling and assay multiplexing for a critically endangered fish’, *Environmental DNA*, (December 2019), pp. 1–11. doi: 10.1002/edn3.64.

**TABLE S2.** Field samples screened for L. lorica and L. nannotis eDNA detection during two sampling events in 2019 (wet and dry season).

**Site eDNA capture method Sample volume (mL) No. field replicates Season**

1 Direct water collection 15 5 Wet

Direct water collection 100 2 Wet

2 Direct water collection 15 5 Wet

Direct water collection 100 2 Wet

3 Direct water collection 15 5 Wet

Direct water collection 100 2 Wet

4 Direct water collection 15 5 Wet

Direct water collection 100 2 Wet

5 Direct water collection 15 5 Wet

Direct water collection 100 4 Wet

Direct water collection 100 5 Dry

Filtration 1,614,000 1 Wet

6 Direct water collection 15 5 Wet

Direct water collection 100 3 Wet

Direct water collection 100 5 Dry

7 Direct water collection 15 5 Wet

Direct water collection 100 4 Wet

Direct water collection 100 5 Dry

Filtration 1,458,000 1 Wet

8 Direct water collection 15 5 Wet

Direct water collection 100 4 Wet

Direct water collection 100 5 Dry

Filtration 1,805,000 1 Wet

**TABLE S2.** continued

**Site eDNA capture method Sample volume (mL) No. field replicates Season**

9 Direct water collection 15 5 Wet

Direct water collection 100 4 Wet

Direct water collection 100 5 Dry

Filtration 1,377,000 1 Wet

10 Direct water collection 15 5 Wet

Direct water collection 100 4 Wet

Direct water collection 100 5 Dry

Filtration 1,145,000 1 Wet

**TABLE S3.** Primer information for *L. lorica* (Litlor_COI) and *L. nannotis* (LnannotisN&P_COI) eDNA assays (from Edmunds, Villacorta-Rath, Huerlimann and Burrows, 2019).

**Primer name Melt temp (˚C) GC content (%) Amplicon size (bp) Oligonucleotide (5' - 3')**

Litlor_COI_F 56.2 47.4 166 CCTGACCGGAATTGTCTTA

Litlor_COI_R 56.4 40.9 GGAGTGTAAAGAGTAACCAGTA

LnannotisN&P_COI_F 60.4 52.4 120 CCGAGCCTATTTTACCTCAGC

LnannotisN&P_COI_R 57.9 47.6 GCTCATAATATAGGTGCGTCC

**TABLE S4.** Percentage of positive detections of the two *Litoria* species from the technical replicates of the 100 mL sampling method at each site, from the wet season and dry season sampling events in 2019.

**Site *Litoria lorica* *Litoria nannotis***

**Wet season Dry season Wet season Dry season**

6 (main pop) 13.9 6.7 44.4 78.3

7 37.5 0 45.8 3.3

8 0* 0* 27.1 0

9 16.7 1.7 ** 1.7

10 12.5 1.7 ** 1.7

* *Litoria lorica* eDNA not expected to be present at this site because there are no upstream populations known on this tributary (see Fig. 1).

** No data for *L. nannotis* due to a mechanical failure of the qPCR machine.

**TABLE S5.** Percentage of positive detections of the two *Litoria* species from field controls and extraction controls (EC) of all eDNA capture methods at each site, from the wet season and dry season sampling events in 2019. “NS” indicates that the site was not sampled at that sampling event.

**Site *Litoria lorica* *Litoria nannotis***

**Wet season Dry season Wet season Dry season**

1 Control 0 NS 0 NS

2 Control 0 NS 0 NS

3 Control 0 NS 0 NS

4 Control 0 NS 0 NS

5 Control 0 NS 0 NS

6 Control 0 0 0 0

7 Control 0 0 0 0

8 Control 0 0 0 0

9 Control 0 0 0 0

10 Control 0 0 0 0

EC 100 mL samples 0 0 0 0

EC 15 mL samples 0 NS 0 NS

EC >1,000 L samples 0 NS 0 NS

**TABLE S6.** Mean Ct values of spiked field samples and MilliQ water to test for qPCR inhibition in samples from the two sampling events in 2019 (wet season and dry season).

**Site Season Ct mean St Error**

**qPCR run 1**

8 (100 mL samples) Wet 26.828 0.052

8 (1,805,000 mL sample) Wet 25.834 0.021

1 (100 mL samples) Wet 26.432 0.026

8 (100 mL samples) Dry 26.913 0.039

Spiked MilliQ water - 26.475 0.030

**qPCR run 2**

3 (100 mL samples) Wet 26.940 0.264

Spiked MilliQ water - 27.152 0.090

**TABLE S7.** Mean Ct values of spiked field controls and MilliQ water to test for qPCR inhibition in samples, from the two sampling events in 2019 (wet season and dry season).

**Site Season Ct mean St Error**

1 Control (100 mL samples) Wet 29.822 1.616

2 Control (100 mL samples) Wet 30.863 0.313

3 Control (100 mL samples) Wet 30.314 0.160

4 Control (100 mL samples) Wet 29.100 0.134

5 Control (1,614,000 mL sample) Wet 31.118 0.053

6 Control (100 mL samples) Wet 30.326 0.219

7 Control (100 mL samples) Wet 30.810 0.130

8 (100 mL samples) Wet 30.252 0.058

9 Control (100 mL samples) Wet 30.377 0.110

10 Control (100 mL samples) Dry 31.015 0.008

Spiked MilliQ water - 30.190 0.090

**FIGURE S1.** Average accumulated monthly rainfall in the sampling area during the year 2019. Red arrows indicate the two sampling events. Source: SILO climate database (<https://www.data.qld.gov.au/dataset/silo-climate-database>). Photos inside the rainfall plot show Site 3 during both sampling events. The top right panel was taken during the wet season sampling (April 2019) and the bottom right panel was taken during the dry season sampling (October 2019). Common features in the photos are numbered on each photo: (1) a live tree; (2) a point on the fallen tree; (3) a particular position on the gravel. Water flow rate was measured during the wet season sampling at Site 1, and estimated at 0.12 m^3^/sec. Flow rate was not calculated during the dry season sampling trip because Site 1 was not accessed and measuring flow at any other site would have not allowed for direct comparisons to the previous sampling event.

**
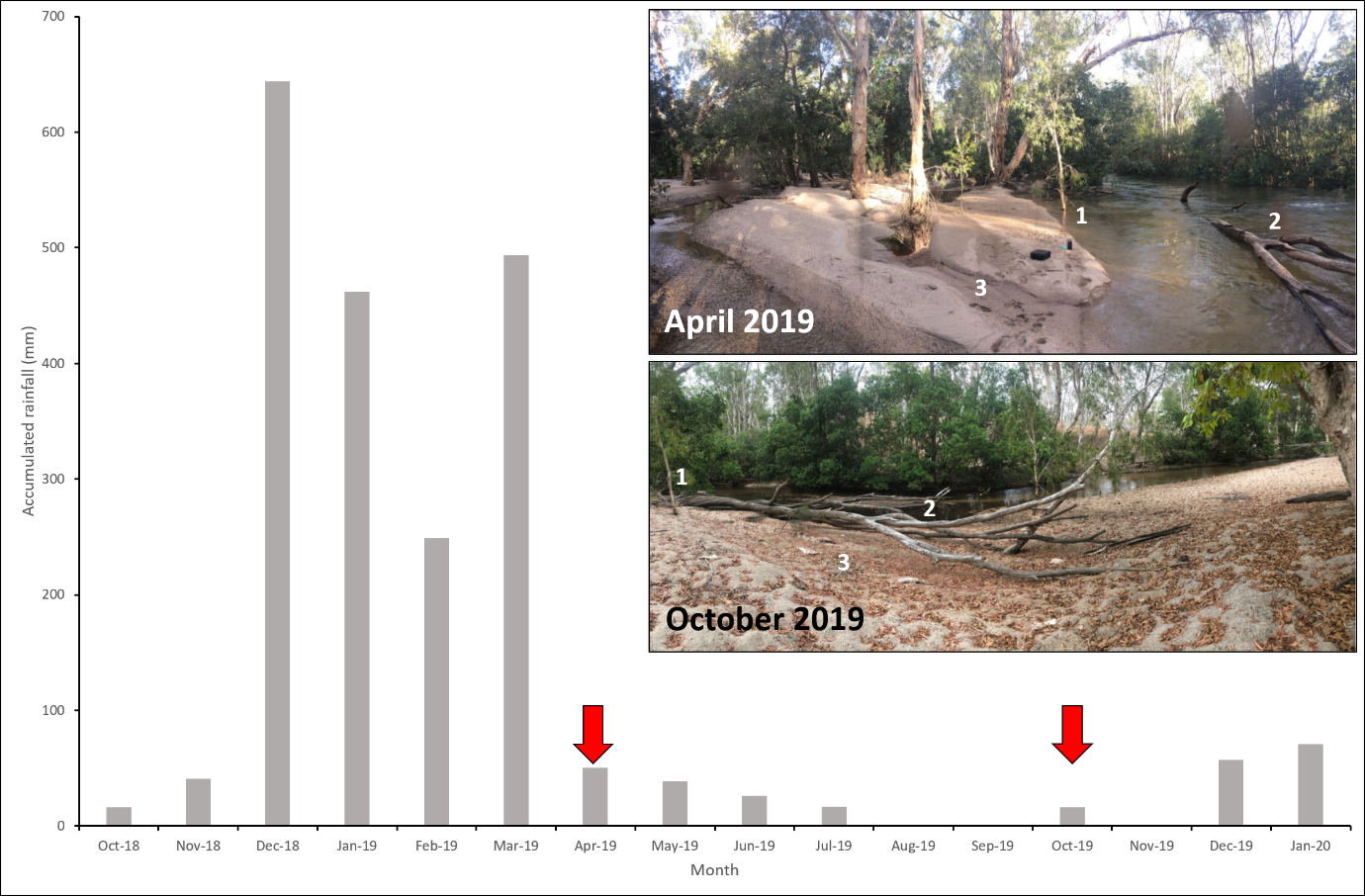
**

**FIGURE S2.** Solutions taken from the Longmire’s preserved filters from the large volume filtration. The numbers on each beaker represent the site number.


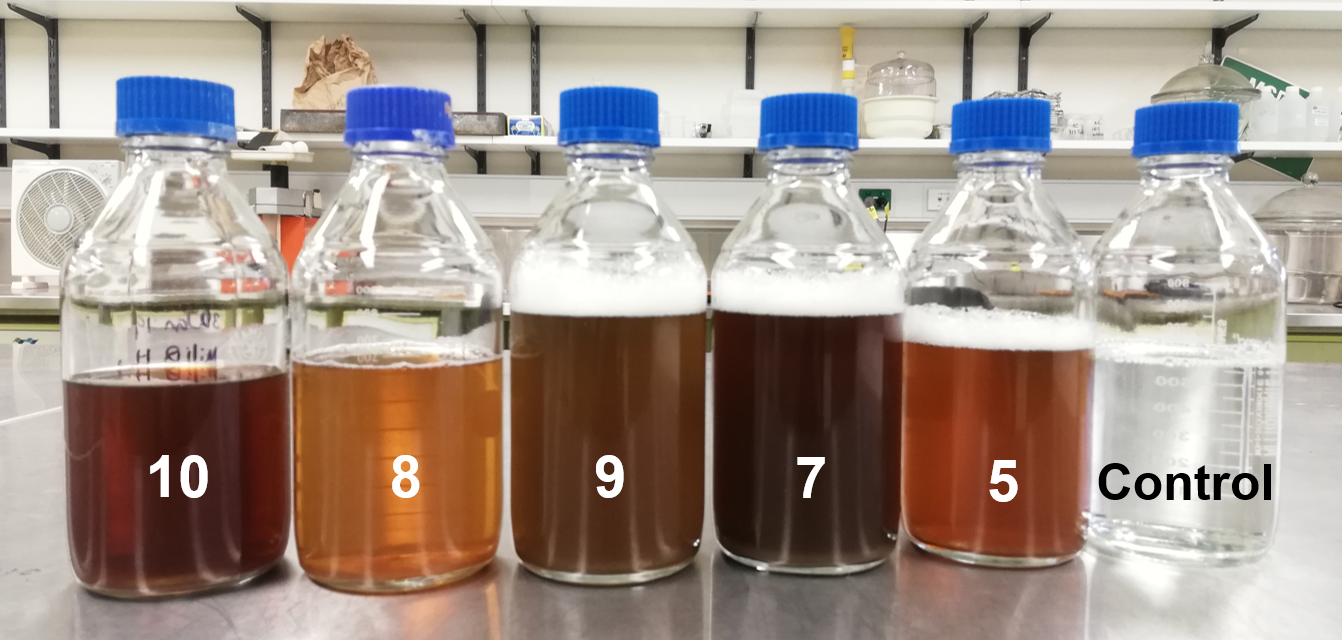

Supplement: Supplemental Information 1 — Tables S1–S7 and Figs. S1 and S2 providing supporting documentation to the article. [file peerj-09-12013-s001.docx]
